# Population-specific design of de-immunized protein biotherapeutics

**DOI:** 10.1371/journal.pcbi.1005983

**Published:** 2018-03-02

**Authors:** Benjamin Schubert, Charlotta Schärfe, Pierre Dönnes, Thomas Hopf, Debora Marks, Oliver Kohlbacher

**Affiliations:** 1 Center for Bioinformatics, University of Tübingen, Tübingen, Germany; 2 Applied Bioinformatics, Dept. of Computer Science, Tübingen, Germany; 3 Department of Cell Biology, Harvard Medical School, Boston, Massachusetts, United States of America; 4 Department of Systems Biology, Harvard Medical School, Boston, Massachusetts, United States of America; 5 SciCross AB, Skövde, Sweden; 6 Quantitative Biology Center, Tübingen, Germany; 7 Faculty of Medicine, University of Tübingen, Tübingen, Germany; 8 Biomolecular Interactions, Max Planck Institute for Developmental Biology, Tübingen, Germany; Fox Chase Cancer Center, UNITED STATES

## Abstract

Immunogenicity is a major problem during the development of biotherapeutics since it can lead to rapid clearance of the drug and adverse reactions. The challenge for biotherapeutic design is therefore to identify mutants of the protein sequence that minimize immunogenicity in a target population whilst retaining pharmaceutical activity and protein function. Current approaches are moderately successful in designing sequences with reduced immunogenicity, but do not account for the varying frequencies of different human leucocyte antigen alleles in a specific population and in addition, since many designs are non-functional, require costly experimental post-screening. Here, we report a new method for de-immunization design using multi-objective combinatorial optimization. The method simultaneously optimizes the likelihood of a functional protein sequence at the same time as minimizing its immunogenicity tailored to a target population. We bypass the need for three-dimensional protein structure or molecular simulations to identify functional designs by automatically generating sequences using probabilistic models that have been used previously for mutation effect prediction and structure prediction. As proof-of-principle we designed sequences of the C2 domain of Factor VIII and tested them experimentally, resulting in a good correlation with the predicted immunogenicity of our model.

## Author summary

Therapeutic proteins have become an important area of pharmaceutical research and have been successfully applied to treat many diseases in the last decades. However, biotherapeutics suffer from the formation of anti-drug antibodies, which can reduce the efficacy of the drug or even result in severe adverse effects. A main contributor to the antibody formation is a T-cell mediated immune reaction caused by presentation of small immunogenic peptides derived from the biotherapeutic. Targeting these peptides via sequence alterations reduces the immunogenicity of the biotherapeutic but inevitably will have effects on structure and function. Experimentally determining optimal mutations is not feasible due to the sheer number of possible sequence alterations. Therefore, computational approaches are needed that can effectively cover the complete search space. Here, we present a computational method that finds provable optimal designs that simultaneously optimize immunogenicity and structural integrity of the biotherapeutic. It relies solely on sequence information by utilizing recent advances in protein *ab initio* prediction and incorporates immunogenicity prediction methods. Thus, the approach presents a valuable tool for bioengineers to explore the design space to find viable candidate designs that can be experimentally tested and further refined.

## Introduction

Protein-based drugs (biotherapeutics) are increasingly used to treat a wide variety of diseases[1, 2]. Although biotherapeutics show high activity and specificity at the initiation of treatment, the gradual build-up of a patient immune response is a bottleneck for even wider usage[3]. The immunogenicity of the biotherapeutic is influenced by multiple factors that can be roughly divided into extrinsic—such as dosage, rout of administration, duration and production impurities—and intrinsic properties like the protein sequence or post-translational modifications [3]. This immune response involves the formation of anti-drug antibodies (ADAs) that target the biotherapeutic itself and cause loss of effect or adverse reactions[3–5]. A prominent example of this adverse effect is in the treatment of hemophilia A (HA) with coagulation Factor VIII, where ADAs develop in 10–15% of all HA patients and as much as 30% of those patients with the most severe form of HA[6]. Patients with the highest need for therapy are thus least likely to benefit. This correlation between severity of the disease and lack of efficacy follows from the fact that the immune system is more likely to recognize the therapeutic Factor VIII as foreign the more severe the natural mutation is, where mutations that cause a total loss of Factor VIII production are most strongly associated with ADA development[7, 8].

The reduction of the immunogenicity has thus become a major step in a the development of a biotherapeutic[5]. The primary focus of reducing immunogenicity has been on humanized monoclonal antibodies (mAbs) that are comprised of foreign complementarity-determining regions in the variable regions, with the remainder of human origin, and, more recently, on fully human mAbs using bioengineering techniques[9, 10]. However, these approaches are not generally applicable to other classes of biotherapeutics and even humanized and full human mAbs can still induce a clinically relevant anti-drug immune response, likely through the CD4^+^ T-cell mediated adaptive immune system[11, 12]. The CD4^+^ T-cell activation is induced by the recognition of linear sequential peptides (called epitopes) derived from the therapeutic protein, which are presented on human leucocyte antigen (HLA) class II molecules of antigen presenting cells. Therefore, the systematic removal of these epitopes by sequence alteration (termed de-immunization) has been successfully used as an alternative approach to reduce the immunogenicity of mAbs and other therapeutic proteins [12–16].

In recent years, computational screening approaches have been developed to suggest protein sequences with reduced overall immunogenicity. The simplest approaches focused solely on introducing point mutations to reduce the amount of CD4^+^ T-cell epitopes by applying well-established epitope prediction methods [17–19]. However, the suggested mutations can have a significant impact on the stability and function of the protein. Naïve approaches not considering the structural impact on the protein will inevitably produce inactive designs. More advanced methods therefore try to exclude potentially harmful mutations by predicting their impact with various metrics [20]. The most recent approaches simultaneous optimize the number of deleted epitopes as well as the stability of the protein approximated either using structural or simple sequence information [21–23].

Recent advances in statistical protein modeling now allow to accurately infer the tertiary structure [24–28] and mutational effects [29–31] of proteins using evolutionary information contained in an multiple sequence alignment of a protein family. The statistical global pairwise entropy model used for protein inference accurately captures co-evolving sites within a protein which can be utilized to identify structural and functional important position using evolutionary couplings (EC) analysis, infer the protein structure, and predicted the effects of mutational changes.

In this work, we present a novel formulation of the de-immunization problem that uses, for the first time to our knowledge, the maximum entropy model for protein design. Incorporating the maximum entropy model, as opposed to force-field based approaches such as FoldX[32] that have been previously used for protein de-immunization, has four distinct advantages: (i) The statistical model does not require a known structure or depend on the conditions in which the structure was measured. (ii) It implicitly considers constraints on residues from interactions with ligands and other proteins, and (iii) models interactions between mutations rather than early filtering of deleterious singles. (iv) A de-immunization approach using the maximum entropy model is likely to generate more viable structures as it minimizes potential damage to protein function at the same time as minimizing the immunogenicity of the biotherapeutic design. The latter can also be achieved by incorporating a force field, such as AMBER[33], into the optimization process [21, 22], which however complicates the de-immunization formulation.

As the frequencies of HLA alleles differ drastically between populations, the immunogenicity of the biotherapeutic differs as well. It is thus imperative to design a biotherapeutic for a specific target population considering their HLA allele frequencies, as opposed to treating each HLA allele equally important during the design process, as all previous methods have done. We therefore developed a new quantitative immunogenicity objective that builds on HLA affinity prediction methods for immunogenicity approximation, as their exists a strong correlation between immunogenicity and HLA binding affinity[34], and considers the HLA allele distributions within different populations. The resulting de-immunization model does not require known structural information about the protein, summarizes functional and structural information that might not be captured by a structure-based model, and considers the varying HLA frequencies in different populations. We also demonstrate how the resulting bi-objective combinatorial optimization problem can be formulated in a concise manner and solved efficiently for relevant problem sizes with a newly developed distributed solving strategy. An experimental validation of the resulting designs confirms that the algorithm can indeed lead to significantly reduced immunogenicity.

## Results

### Formulation of the de-immunization problem

The problem of protein de-immunization can be described as identifying amino acid substitutions that reduce immunogenicity by removing T-cell epitopes while at the same time keeping the structure and function of the protein intact. We therefore define the problem of protein de-immunization as a bi-objective optimization problem. The first objective characterizes the immunogenicity of the target protein with respect to a set of HLA alleles. The immunogenicity objective *I*(*S*|*H*,*P*_*H*_) combines the immunogenicity of each predicted epitope over a certain binding threshold weighted by the HLA allele frequencies *P*_*H*_ of a specific target population represented by their prevalent HLA alleles *H* [35]. The second objective *E*(*S*) approximates the protein fitness via the statistical energy of the protein sequence, computed by the pairwise maximum entropy model inferred using a multiple sequence alignment (MSA) of the target protein family[24–28].

More formally, we define the protein de-immunization problem as follows: Given a protein sequence S of length n and a set M_i_ of possible alterations per position 1 ≤ i ≤ n. We seek a mutant S′ of S with k alterations for which S′[i] ∈ M_i_ ∀ 1 ≤ i ≤ n holds and that minimizes:

$$\begin{array}{l} {argmin}_{S^{\prime }}\;(I(S^{\prime }|H,P_{H}),-E(S^{\prime }))\\ s.t.\;S\prime \in Χ, \end{array}$$

The model therefore optimizes the tradeoff between these two objectives and produces a set of Pareto-optimal designs of the protein sequence.

### Immunogenicity objective function definition

The first objective of the de-immunization model is an adaptation of the immunogenicity score introduced by Toussaint *et al*. for epitope selection in the context of *in silico* vaccine design and is defined as follows [35]:

$$I(E|H,P_{H})=\sum_{e\in A}\sum_{h\in H}p_{h}∙i_{e,h},$$

with *A* being a set of epitopes, *i*_*e*,*h*_ the immunogenicity of epitope *e* ∈ *A* bound to HLA allele *h* ∈ *H*. It assumes that each epitope independently influences the immune response with respect to all considered HLA alleles. The contribution of an HLA allele *h* ∈ *H* is directly proportional to its probability *p*_*h*_ of occurring within the target population *H*.

### Protein fitness objective function definition

The second objective is an evolutionary statistical energy of sequences computed by a pairwise maximum entropy model of protein families. Under these family-specific models, the probability for a protein sequence (X_1_,…, X_n_) of length *n* is defined as

$$P\left(X_{1},\ldots X_{n}\right)=\frac{1}{Z}e^{-E\left(x\right)}$$

$$E\left(X\right)=\sum_{1\leq i<j\leq n}J_{ij}\left(X_{i},X_{j}\right)+\sum_{1\leq i\leq n}h_{i}\left(X_{i}\right)$$

where the pair coupling parameters *J*_*ij*_*(X*_*i*_, *X*_*j*_*)* describe evolutionary co-constraints on the amino acid configuration of residue pairs *i* and *j* for all amino acids and the parameters *h*_*i*_*(X*_*i*_*)* corresponds to single-site amino acid constraints. The partition function *Z* is a global normalization factor summing over all possible amino acid sequences of length *n* [24, 25]. The parameters J_ij_ and h_i_ are inferred from a protein family sequence alignment using an iterative approximate maximum likelihood inference scheme (pseudo-likelihood maximization) under l_2_-regularization to prevent overfitting. Given an inferred probability model for a family, the statistical energy −*E*(*X*) can be used to quantify the fitness of specific sequences. Recent work has demonstrated that changes of E(X) quantitatively correspond to the experimental phenotypic consequences of mutations, including effects on protein stability and organismal growth [31]. To maintain protein function while minimizing immunogenicity, the second objective function is defined as the minimization of −*E*(*X*) given inferred parameters *h*_*i*_ and *J*_*ij*_ from a multiple sequence alignment of the biotherapeutic.

Evolutionary coupling (EC) strength between pairs of positions *i* and *j* is computed using the Frobenius norms of the matrices J_ij_ with subsequent correction for finite sampling and phylogenetic effects (average product correction)[27]. The evolutionary couplings are predictive of residue proximity in many protein families, and the cumulative score of one position to all others (EC enrichment score) is indicative of functionally and structurally important positions [27].

### Derivation of the integer linear program representation of the de-immunization model

We solve the stated de-immunization problem as a bi-objective mixed integer linear program (BOMILP). Solving a BOMILP finds all Pareto-optimal solutions to linear objectives with affine constraints and additional integrality constraints on a subset of the variables. The model is based on Kingsford *et al*.’s ILP formulation of the side-chain placement problem [36]. But instead of selecting energetically favorable rotamers, we encode each state of the model as a possible amino acid substitution at each position. A binary decision variable *x*_*i*,*a*_ for each position *i* ∈ {1..*n*} and each possible variation *a* ∈ *M*_*i*_ is introduced with *x*_*i*,*a*_ = 1 if this variant will be part of the final mutant *S*′. An additional binary variable is introduced for each pair of variants and positions notated *w*_*i*,*j*,*a*,*b*_ with *w*_*i*,*j*,*a*,*b*_ = 1 if variant *a* at position *i* and variant *b* at position *j* have been selected as part of the solution *S*′. These variables are associated with their inferred fitness terms *h*_*i*,*a*_ and *J*_*i*,*j*,*a*,*b*_ to form the second objective function (Table 1 O2).

**Table 1:** Bi-objective integer linear program formulation of the de-immunization problem.

Definition:	
Objectives:	
(O1) $min_{x,w}\;\sum_{h\in H}p_{h}∙\sum_{i=1}^{n-e_{n}}max(0,(\sum_{j=0}^{e_{n}-1}\sum_{a\in M_{i+j}}x_{i+j,a}∙\phi (h,a,j))-\tau_{h})$	
(O2) $min_{x,w}\;\sum_{i=1}^{n}{x_{i,a}∙h}_{i,a}+\sum_{i=1}^{n}\sum_{1\leq i<j\leq n}{w_{i,j,a,b}∙J}_{i,j,a,b}$	
**Constraints:**	
(C1) $\sum_{a\in M_{i}}x_{i,a}=1$	∀ i ∈ {1..2}
(C2) $\sum_{b\in M_{j}}w_{i,j,a,b}=x_{i,a}$	∀ i, a ∈ Mi, *i* > *j* ∈ {1..n}
(C3) $\sum_{a\in M_{i}}w_{i,j,a,b}=x_{j,b}$	∀ j, b ∈ Mj, *j* > *i* ∈ {1..n}
(C4) $\sum_{i=1}^{n}\sum_{a\in W_{i}}(1-x_{i,a})\leq k$	

The immunogenicity objective, in contrast to the problem formulation of Toussaint *et al*., in which the immunogenicity of each candidate epitope *e* ∈ *A* could be pre-calculated, does not have an easy ILP representation. Prediction methods must be directly incorporate into the ILP to approximate the immunogenicity of the current mutant *S*′. Therefore, we use TEPITOPEpan [37], a linear HLA-epitope affinity prediction method, as internal prediction engine since it has been demonstrated to have good predictive power and can be easily integrated into the ILP framework. More advanced, potentially non-convex non-linear prediction models such as artificial neural networks cannot readily be integrated into the problem formulation as the resulting optimization problem would be discreet, non-convex, and non-linear. This class of optimization problems is known to be hard to solve to optimality even for small instances [38] and thus out of reach for design problems of relevant size.

With linear (matrix-based) methods, the integration is possible by scoring each peptide generated with a sliding window of width *e*_*n*_ for each allele *h* ∈ *H* independently by summing over TEPITOPEpan’s position specific scoring matrix $\Phi (h,a,j)\to R_{\geq 0}$ for amino acid *a* ∈ *M*_*i*+*j*_ at position *i* + *j* with *i* ∈ {1..(*n* – *e*_*n*_)} and *j* ∈ {0..(*e*_*n*_ − 1)}. To only consider predicted binding epitopes, the binding threshold *τ*_*h*_ of each HLA allele *h* ∈ *H* is subtracted from the sum score of an epitope and embedded into a hinge loss function. The summarized contribution of an allele *h* ∈ *H* is than weighted by its population probability *p*_*h*_. To make the prediction scores comparable across HLA alleles, the position specific scoring matrices of TEPITOPEpan were z-score normalized and the binding thresholds adjusted accordingly. The final immunogenicity score consists of the sum of all allele-wise weighted sums (Table 1, O1).

To construct a consistent model, three constraints have to be introduced guaranteeing that only one amino acid per position is selected (Table 1, C1) and that only pairwise interactions are considered for selected variants (i.e., *J*_*i*,*j*,*a*,*b*_, = 1 ↔ *x*_*i*,*a*_ = 1 ⋀ *x*_*j*,*b*_ = 1, see Table 1 C2 and C3). Constraints C2 and C3 can be further relaxed by dividing the pairwise fitness values into positive and negative sets [36], which is done in practice but disregarded here for ease of presentation. To be able to restrict the mutant to a specific number of introduced variations, constraint C4 limits the number of deviating amino acids to the wild type sequence *W*. A detailed formulation of the complete optimization problem can be found in S2 Material.

### Computational and experimental evaluation

As proof-of-principle, we tested the ability of the model to find low immunogenic constructs of the C2 domain of Factor VIII as the domain is highly immunogenic and involved in the ADA development in hemophilia A patients when used therapeutically [39, 40].

### Evolutionary couplings accurately predict structure and mutational effects

Evolutionary couplings computed from sequence alignments have been used successfully to predict the phenotypic effects of mutations [29–31, 41], as well as the 3D structure shown in earlier work [24–28]. The approach assigns an evolutionary statistical energy to any protein sequence that is hypothesized to correspond to the fitness of the molecule. The computation of the statistical energy of the protein and any changes to it after mutation is automatic and does not depend on computing or knowing the 3D structure. Therefore, we reasoned that we could use this statistical model in a generative mode for design within the algorithmic de-immunization process.

**Fig 1:**
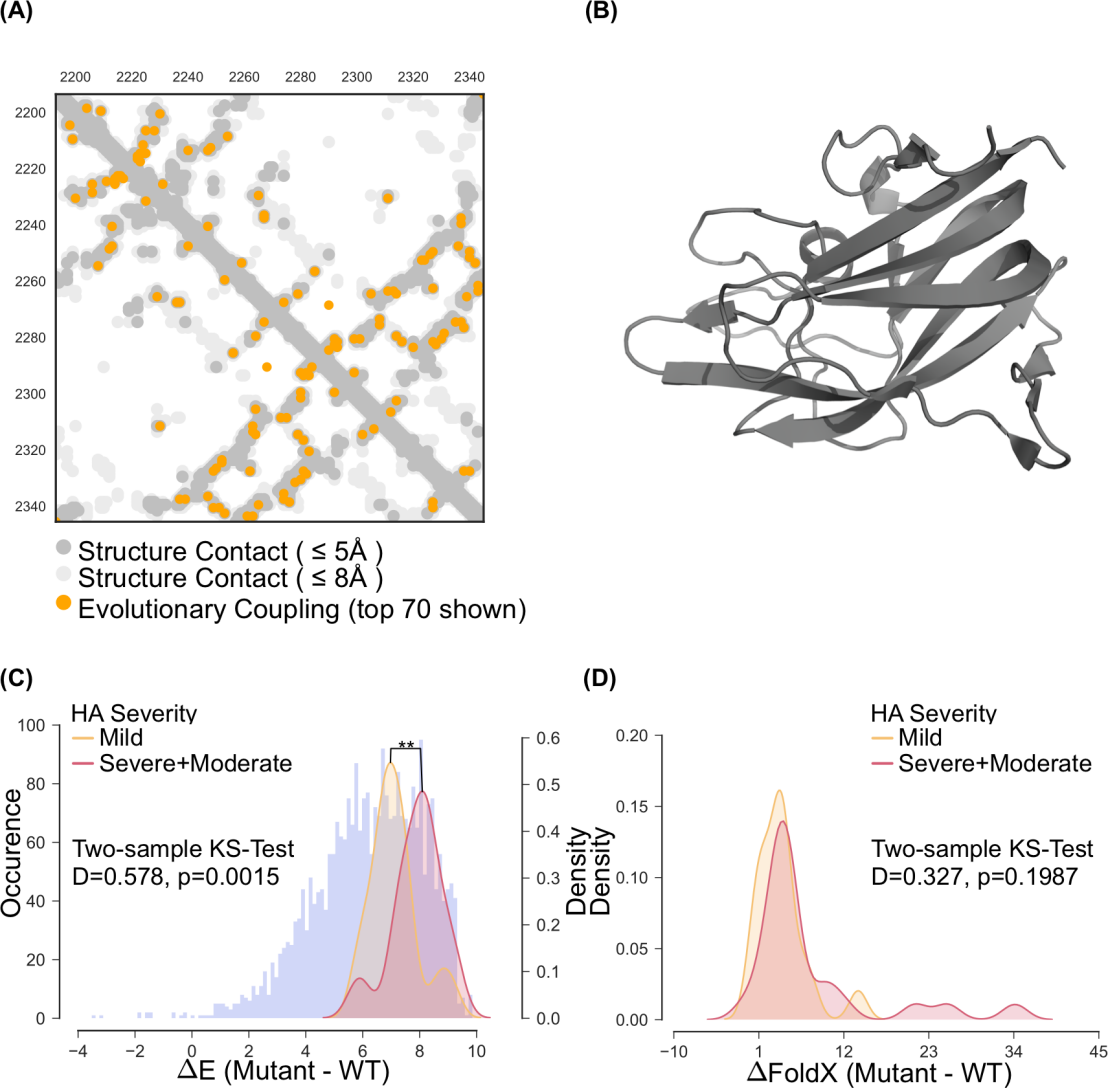
(A) Contact map of Factor VIII’s C2 domain. The gray circles represent the known crystal structure (pdb: 3hny), while the orange dots represent predicted evolutionary couplings. (B) The tertiary structure of Factor VIII’s C2 domain (pdb: 3hny). (C) Statistical energy/fitness change density separated by HA severity status (red and yellow) and the complete maximum entropy landscape of single point mutations (blue). As expected, all known single point mutations with known HA severity status reside in the lower percentile of the energy landscape. The severe and moderate HA cases are clearly separable from the mild cases using the maximum entropy model prediction. In contrast, the two distributions cannot be clearly separated using FoldX predictions (D).

Previous work on predicting the effect of mutations suggested that the model accuracy depends on the diversity of the sequence alignment and the ability to predict the 3D structure accurately[31]. We used the precision of the total epistatic constraints between residue pairs as an approximation of the model validity. Overall, 70 long-range evolutionary coupled residue pairs (ECs) have a probability of at least 90% of being significant and 65 of these (93% precision) are close in space (less than 5Å; Fig 1A, Supplementary S1 Material) in a 3D structure of Factor VIII’s C2 domain (pdb: 3hny[42]; Fig 1B).

To assess how well maximum entropy model can predict the effects of specific mutations compared to force-field methods, we used our maximum entropy model and FoldX predictions in a multinomial and logistic regression to predict hemophilia A severity (severe, moderate, and mild) based on patient data collected from the Factor VIII variant database (http://www.factorviii-db.org, Supplementary S1 Table). Since the severity of HA is directly correlated with instability and malfunctioning of Factor VIII, the prediction of disease severity can be seen as a proxy for functional and structural effect prediction. The multinomial regression model, using the change in statistical energy between mutant and wild type as independent variable, shows a moderate ability to predict the clinical outcome (F1-micro of 0.65 ± 0.09, F1-macro of 0.47 ± 0.07).

The performance of a FoldX-based multinomial regression model however was significantly worse (one-sided Wilcoxon signed rank test, V = 13558, p-value < 2.2e-16; F1-micro of 0.49 ± 0.11, F1-macro of 0.35 ± 0.09). We combined the severe and moderate clinical classes and performed a logistic regression, which improved the prediction performance of our maximum entropy model (weighted AUC of 0.72 ± 0.11, weighted F1-score of 0.73 ± 0.11; Supplementary S1 Table; Fig 1C). The FoldX-based logistic regression model was again outperformed by our maximum entropy model (one-sided Wilcoxon signed rank test, V = 15633, p-value < 2.2e-16; Fig 1D), but also yielded higher predictive power compared to its multinomial model (weighted AUC: 0.62 ± 0.11, weighted F1 0.58 ± 0.13).

### Strong immunogenic region contains functional sites

We first *in silico* identified a narrow region of high immunogenic potential for the three most prevalent HLA alleles in the European population (DRB1*15:01, DRB1*03:01 and DRB1*07:01; accounting for 70% of the patients in Western Europe) to facility experimental evaluation.

**Fig 2:**
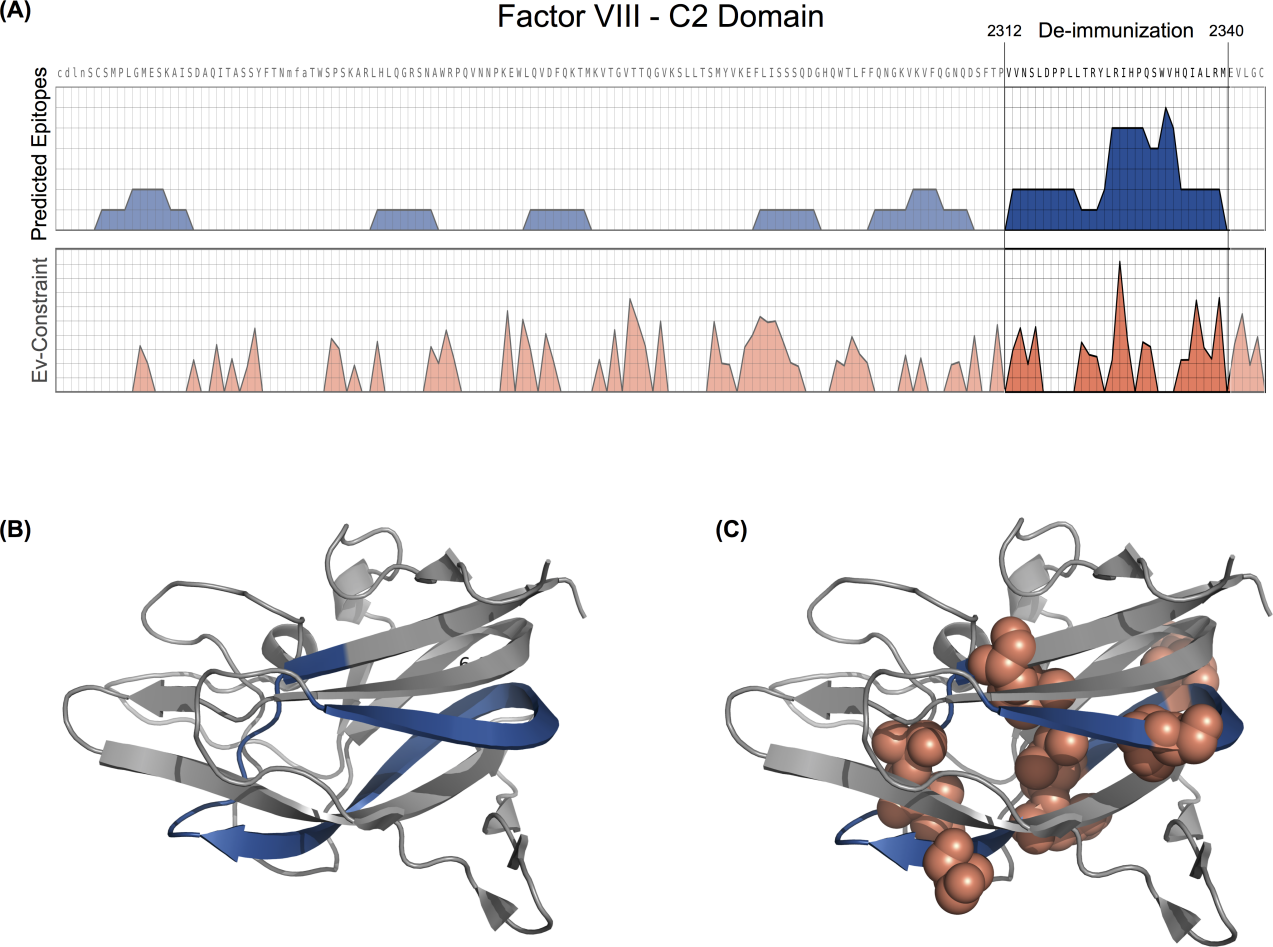
(A) Immunogenicity screening for three DRB1 alleles (DRB1*15:01, DRB1*03:01 and DRB1*07:01) with TEPITOPEpan. All peptides who’s predicted binding affinity fell into the 95% percentile of TEPITOPEpan’s score distributions have been considered epitopes. The blue regions depict the cumulative number of predicted epitopes per position and the orange regions depict the EC cumulative summary scores of top 70 ECs. Six immunogenic regions can be identified based on the *in silico* screening, with region 2,321 to 2,340 having the highest number of overlapping epitopes (9 out of 16 predicted epitopes) and was thus chosen as de-immunization target. It is comprised of the highest evolutionary coupling pairs. (B) The tertiary structure of Factor VIII’s C2 domain of with highlight immunogenic region selected for de-immunization redesign in blue. (C) The tertiary structure of Factor VIII’s C2 domain with marked top eight EC (red spheres) that coincide with the identified immunogenic region (blue).

We screened the C2 domain of Factor VIII using TEPITOPEpan [37] for peptides binding to the three HLA alleles. Each peptide that fell into the 95% percentile of TEPITOPEpan’s score distribution of an HLA molecule was considered an epitope. We predicted 16 epitopes in 6 regions of the C2-domain of Factor VIII (UniProt: FA8_HUMAN). The region with the highest scoring immunogenicity (residues 2,312–2,340) had nine of the 16 predicted strong binding epitopes (Fig 2A and 2B), making it a prime candidate region for de-immunization design. However, there is evidence that this very region might be of high functional importance for the protein; The region is enriched for conserved co-variation of residues and contains a known membrane-binding motif [42]. Eight of the top ten evolutionary couplings involve residues in the high immunogenic region (Fig 2C). In general, the region is enriched for strong evolutionary couplings (sign test, s = 124, n = 130, p-value < 2.2e-16, CI95 = [0.90, 0.98]; Fig 2A). Hence there is a risk that mutations designed to minimize immunogenicity could be detrimental to protein function and the method we have developed here is specifically designed to minimize the risk of both.

### De-immunization of Factor VIII’s C2 domain

**Fig 3:**
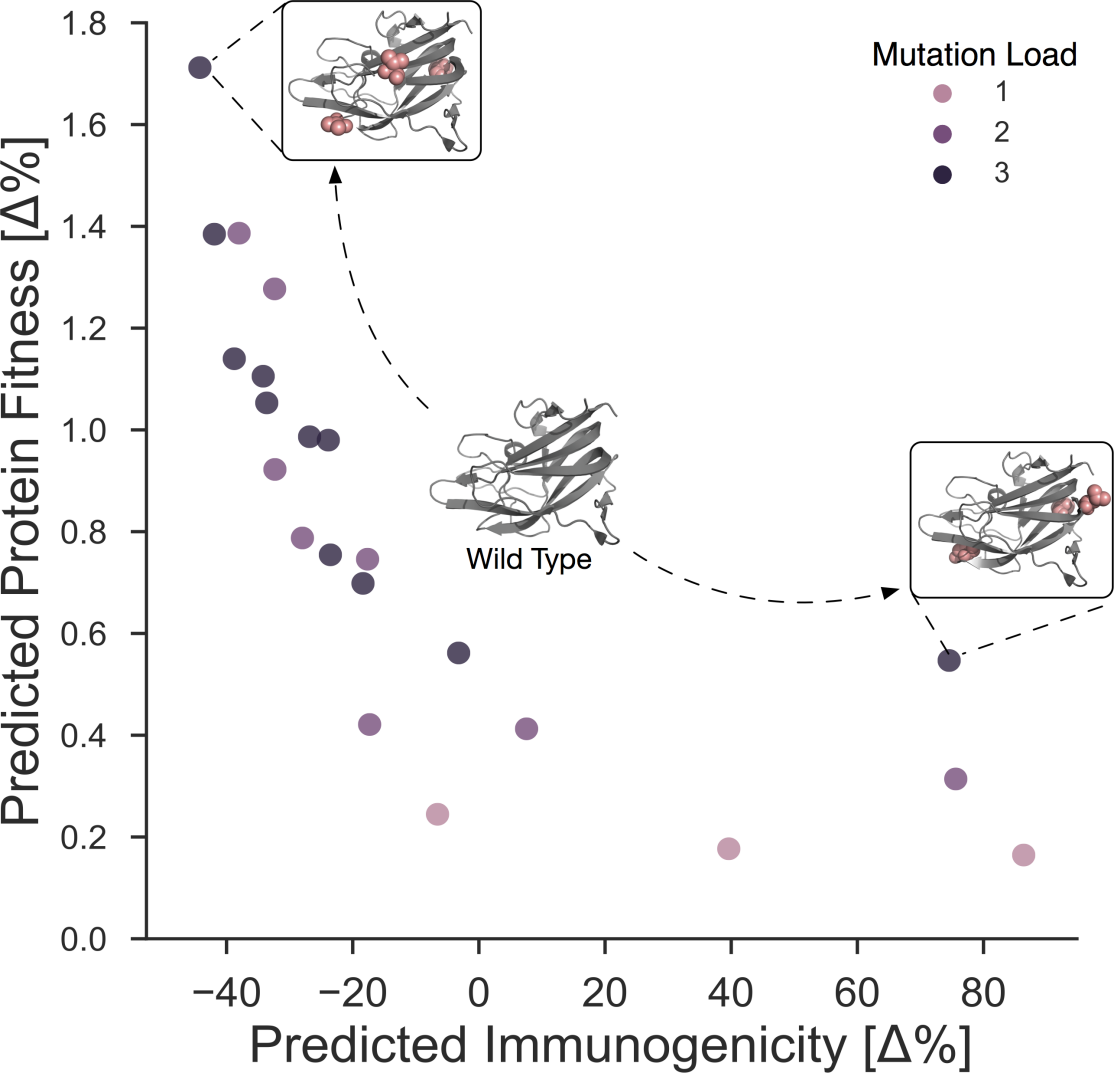
Pareto front of de-immunized designs in percent change compared to the wild type with k = 1,2,3 mutations. Each design is a tradeoff between the immunogenicity and the protein fitness function and represents a new sequence (here represented as tertiary structures). Immunogenicity is approximated by the HLA binding affinity predictions for a set of HLA molecules weighted by their HLA allele frequency in a specific population. The red spheres within the tertiary structures mark the mutated residues.

We next solved our bi-objective mixed integer de-immunization model to design sequences of the identified highly immunogenic region resulting in 21 Pareto-optimal sequences with up to three simultaneous point mutations (Table 2, Fig 3). Although the model was set up to constrain sequence substitutions solely to the identified immunogenic region, the resulting fitness change was optimized based on the interactions with all sites in that protein domain.

**Table 2:** De-immunization results for mutation loads of *k* = 1,2,3.

ID		Mutation	Epitopes	*Δ*I	*Δ*E	*Δ*FoldX
wt			16			
0	V2333E		11	-0.38	1.14	0.95
1	L2321F		16	2.25	0.83	0.7
2	Q2335H		16	4.91	0.77	0.07
3	Y2324L,V2333E		9	-2.16	6.47	0.56
4	Y2324H,V2333E		10	-1.84	5.96	3.78
5	R2326K,V2333E		10	-1.84	4.31	2.67
6	L2321T,V2333E		10	-1.59	3.68	2.13
7	L2321Y,V2333E		11	-1.01	3.48	1.7
8	L2321F,V2333E		12	-0.99	1.97	1.66
9	V2333E,Q2335H		12	0.43	1.93	1
10	L2321F,Q2335H		17	4.3	1.47	0.57
11	V2313M,Y2324L,V2333E		8	-2.52	7.99	0.16
12	L2321T,I2327L,V2333E		8	-2.39	6.47	3.1
13	L2321F,R2326K,V2333E		10	-2.21	5.32	3.36
14	V2313M,L2321T,V2333E		9	-1.95	5.16	1.61
15	L2321F,I2313V,V2333E		10	-1.92	4.92	3.29
16	L2321F,I2313L,V2333E		10	-1.53	4.61	2.78
17	V2313T,L2321F,V2333E		10	-1.36	4.58	1.49
18	V2313M,L2321F,V2333E		11	-1.34	3.52	0.99
19	L2321F,Y2324F,V2333E		12	-1.05	3.26	0.93
20	L2321F,V2333E,Q2335H		13	-0.19	2.62	1.44
21	L2321F,Y2324F,Q2335H		17	4.24	2.55	0.21

Even though none of the 21 designed sequences were predicted “fitter” than the wild–type, they were all close to the wild-type fitness. The computed fitness of 20 out of 21 designs resided in 95% percentile or higher when compared to the whole distribution of single, double, and triple mutations, suggesting that the protein would remain stable and functional. The sequence with the highest difference to wild-type fitness prediction (Design-11; V2313M, Y2324L, V2333E) was in the 90% percentile and still close to WT fitness (reduction of 1.7%). It exhibited also the maximal predicted reduction of immunogenicity (immunogenicity reduction of 45%) deleting eight out of nine epitopes of the identified region. The next-best triple mutant (Design-12; L2321T, I2327L, V2333E) resulted in the deletion of eight epitopes with an immunogenicity reduction of 42% and a fitness reduction of 1.28%.

**Fig 4:**
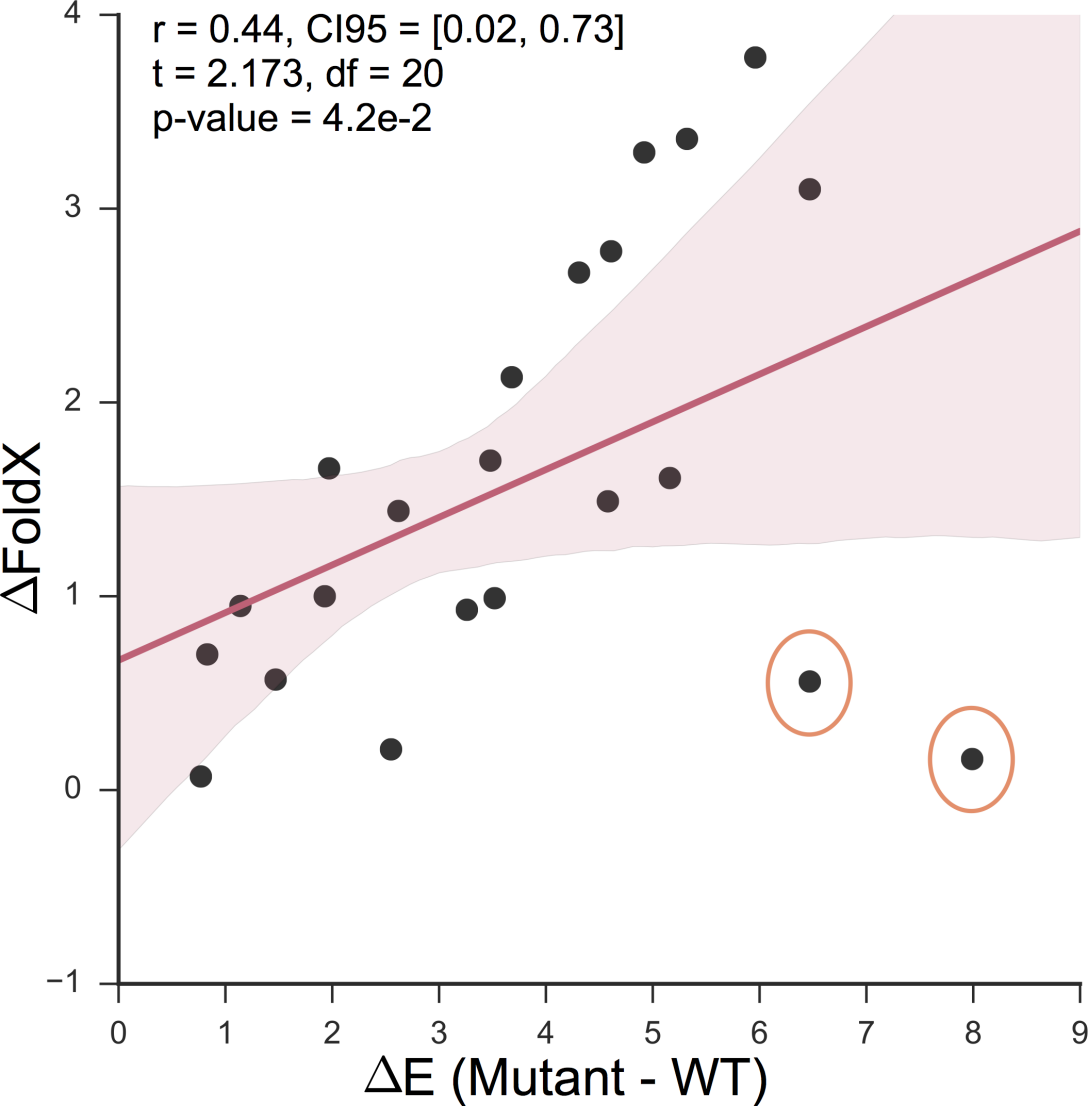
Evolutionary couplings-based model and FoldX prediction correlations. The red line is a fitted linear regression, and the red tube represents its 95-confidence interval. The orange-circled dots are the two mutational designs with the highest discrepancy. FoldX predicted these two mutations less deleterious compared maximum entropy model, although both designs introduced a mutation at a membrane-binding site.

Previous work that aims to increase the likelihood of a functional protein after mutation design, has used force-field based modeling, such as FoldX [32]. As to distinguish the differences between FoldX and the employed maximum entropy model we predicted the mutation effects of the 21 designs with the EV model and FoldX (Table 2). The predictions of the maximum entropy model only moderately correlate to those using FoldX (r = 0.44, CI95 = [0.02, 0.73], t = 2.173, df = 20, p-value = 4.2e-2; Fig 4). The two most deviating mutations between the two prediction methods were Design-11 (V2313M, Y2324L, V2333E) and Design-3 (Y2324L, V2333E), both of which introduced a mutation at a membrane-binding site[42]. FoldX predicted these designs comparatively less deleterious than the predictions of the maximum entropy model. One explanation for this discrepancy is that it would be harder for force-field based methods to capture the membrane binding constraints unless they were in the structure used.

### Experimental validation

**Fig 5:**
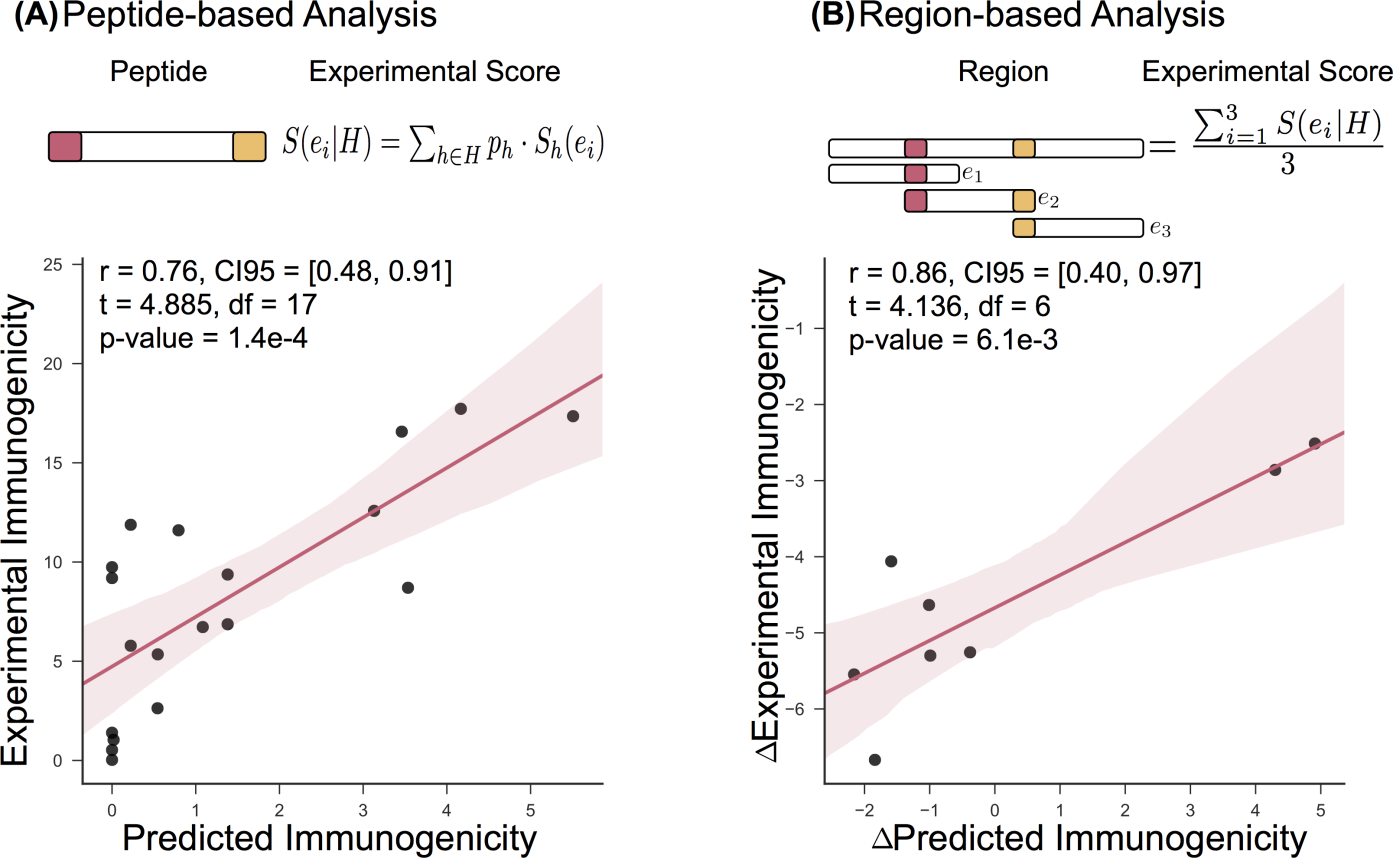
(A) Correlation of experimental and predicted immunogenicity of each peptide. The experimental immunogenicity score of a peptide is defined as the linear combination of the individual experimentally determined relative HLA binding affinity of each HLA allele h ∈ H weighted by the HLA allele frequency. (B) Correlation of experimental and predicted immunogenicity of the whole redesigned region. The summarized immunogenicity score of the whole region is the linear combination of the overlapping peptides used to reconstruct the region, normalized by total number of peptides used. The predicted immunogenicity scores per peptide were computed according to our immunogenicity objective function. The red lines are a fitted linear regression and the red tubes represent their 95-confidence interval.

To test the designs, we synthesized twenty overlapping 15-mer peptides containing the introduced mutations and their wild-type counterparts. The peptides maximally covered the predicted epitopes around the mutations of all designed constructs that contained one and two mutations (Supplementary S2 Table). The affinity of these peptides to the three HLA alleles was measured at time zero and after 24 hours (Methods, Supplementary S3 and S4 Tables). We linearly combined the measured relative affinity scores across HLA alleles weighted by their allele frequencies for each peptide respectively to produce a score that approximates the overall immunogenicity (Fig 5A). Results are presented for measurements taken at time point zero. Measurements made after 24 hours were very similar (r = 0.94, CI95 = [0.86, 0.98], t = 11.81, df = 17, p-value = 1.3e-09) and thus resulted in similar correlations. Overall, the measured and predicted immunogenicity of the tested peptides correlated well with r = 0.76 (CI95 = [0.48, 0.91], t = 4.885, df = 17, p-value = 1.4e-4; Fig 5A). Next, we compared the predicted and measured gain or loss in immunogenicity for the whole region by reconstructing the targeted region using overlapping peptides. The measured relative scores were linearly combined (as previously) and then normalized to the number of overlapping peptides used in the reconstruction (Fig 5B). The difference in the measured scores between the wild type and mutant regions can be thought of as a proxy for gain or loss in immunogenicity and correlated well to the predicted changes (r = 0.86, CI95 = [0.40, 0.97], t = 4.136, df = 6, p-value = 6.1e-3; Fig 5B).

## Discussion

This work introduced a novel method to reduce a protein’s immunogenicity while maintaining its structural integrity requiring only sequence information of the target protein. The method uses a different immunogenicity objective compared to all previous approaches, accounting for both relative epitope strength and HLA allele frequency information of a target population. The HLA distribution can differ tremendously between populations influencing the immunogenicity of a protein and hence the design process should account for the difference by prioritizing different T-cell epitopes. We further combined these objective functions in a bi-objective mixed-integer linear program and introduced a novel solving strategy that guarantees to find the full and exact Pareto front of our de-immunization model. While guaranteeing global optimality, an integer linear program imposes constraints on the functional form the immunogenicity and protein fitness objectives can take. Only linear or simple convex functions can be integrated into an integer linear program, thus prohibiting the use of non-linear, non-convex prediction models. However, integrating such complex, non-convex methods would lead to a highly complex optimization problem that is effectively impossible to solve to optimality for design problems of relevant size.

The fact that the highly immunogenic region, *a priori* identified during an *in silico* screening and independently described by others [19], coincides with a highly evolutionary connected as well as functionally important region underlines the need for methods that are capable of incorporating functional and structural integrity prediction in the de-immunization process. The de-immunization model introduced demonstrates the power of such approaches. The predicted immunogenicity of the complete domain could be reduced by 45% without disrupting the fitness landscape extensively. Moreover, the observed highly significant correlations between measured and predicted immunogenicity both on individual peptide and (reconstructed) segment level affirmed that the underlying assumptions made by the model are sufficient to predict the influence of mutation in terms of immunogenicity.

In the case of this Factor VIII domain, we found no advantage to structure and force-field base approaches to assessing the effect of clinical mutation classification; structure-based approaches may even be a disadvantage when structure information is incomplete (e.g., binding partners not present). This suggests that sequence information may be sufficient for de-immunization design, and is consistent with the previous observation that sequence alignments can be used to identify constrained interacting residues across biomolecules as well as the effect of mutations [31, 43]. However, high-quality diverse sequence alignments are not always available, especially for chimeric or synthetic proteins.

In summary, we proposed a novel de-immunization model that integrates quantitative immunogenicity optimization with sequence-based fitness optimization and used the approach to design novel C2 domains of Factor VIII that can be further validated for clinical application using mouse models or T-cell proliferation assays based on PBMCs of HA patients. The approach will allow bioengineers to reliably explore the design space of the target protein to select promising candidates for experimental evaluation.

## Methods

### Pre-processing

To reduce the search space, a filtering approach based on position specific amino acid frequency *f*_*i*_(*a*) (i.e., conservation) can be applied. Only amino acids at position *i* ∈ {1..*n*} exceeding a certain frequency threshold *ζ* are considered as possible substitution at a site. Hence, the set of possible substitutions per position is defined as *M*_*i*_: = {*a* ∈ *Σ*|*f*_*i*_(*a*) ≥ *ζ*}. The wild type amino acid is additionally added if it does not exceed the frequency threshold. This filtering assumes that variants that are not or infrequently observed are harmful due to either destabilizing effects, reduction of function, or intervening effects with interaction partners.

### Solving a bi-objective integer program

Special strategies must be applied to solve a BOMILP. Popular methods to solve discrete multi-objective problems include the *ε*-constraint [44], perpendicular search [45], and the augmented weighted Chebychev method [46]. All have their own limitations. The recently published rectangle splitting approach tries to overcome these [47, 48]. For solving the de-immunization problem, we developed a parallel two-phase version of the rectangle-splitting approach that can exploit the parallel nature of the algorithm and can effectively utilize modern distributed computing resources. In the following we sketch the newly developed two-phase approach.

First, we introduce necessary notations and concepts (adopted from Boland *et*. *al*.). Let $z^{1}=(z_{1}^{1},z_{2}^{1})$ and $z^{2}=(z_{1}^{2},z_{2}^{2})$ be two points in solution space with $z_{1}^{1}\leq z_{1}^{2}$ and $z_{2}^{2}\leq z_{2}^{1}$. Further we define *R*(***z***^1^,***z***^2^) to be the rectangle spanned by ***z***^**1**^ and ***z***^**2**^. A nondominated point within *R*(***z***^1^,***z***^2^) can be found with the following sequential operation (see proof in[47]):

$$\begin{array}{l} (1)\;\bar{z_{1}}=\underset{x\in \chi }{\min}\;z_{1}(x)\\ s.t:\;z(x)\in R(z^{1},z^{2})\\ (2)\;\bar{z_{2}}=\underset{x\in \chi }{\min}\;z_{2}(x)\\ s.t:\;z(x)\in R(z^{1},z^{2})\;and\;z_{1}\leq \bar{z_{1}} \end{array}$$

These operations will be denoted as $\tilde{z}=lex\;\min_{x\in X}\;\left\{z_{1}(x),z_{2}(x):z(x)\in R(z^{1},z^{2})\right\}.$

**Fig 6:**
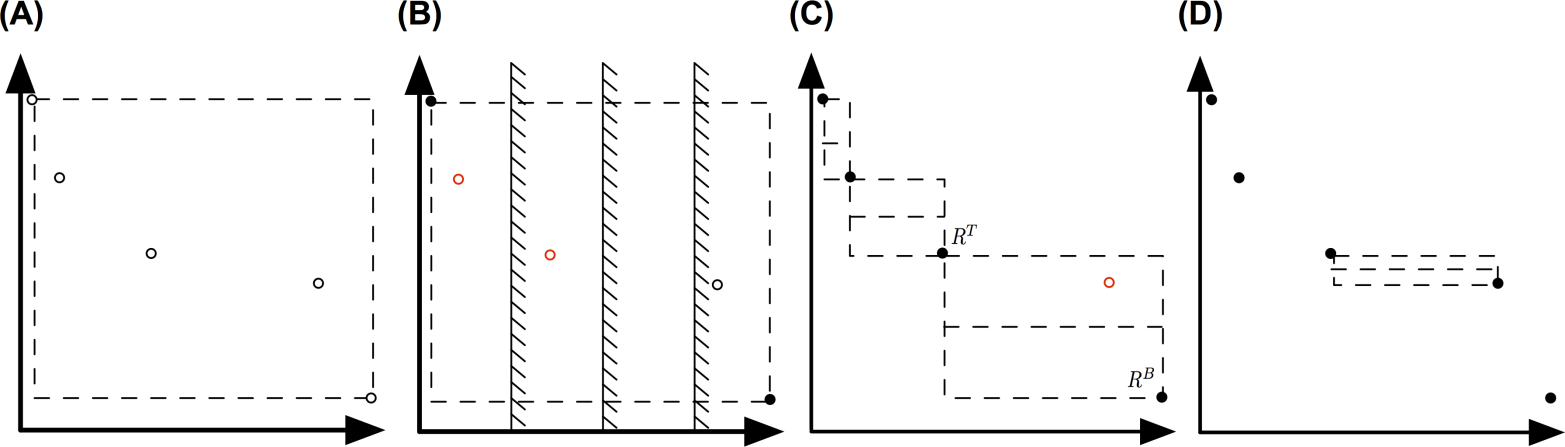
Depiction of the parallel two-phase rectangle splitting approach. (A) First, the boundaries of the Pareto front are identified. (B) Then, the space between the boundaries is evenly divided and searched in parallel for nondominated points using the ε-constraint method. (C) The identified nondominated points are used to initiate rectangle search spaces which can be processed in parallel using the standard rectangle-splitting approach, by splitting the rectangle in half and searching independently the bottom and top half (D). If the corner points of the rectangles are found during the search, it is proofs, that no further nondominated point resides within the search space and all points have been identified.

As a first step of the two-phase parallel rectangle-splitting approach the boundaries of the Pareto front are calculated by solving

$$z^{T}=lex\;\underset{x\in X}{\min}\left\{z_{1}(x),z_{2}(x):z(x)\in R((-\infty ,\infty ),(-\infty ,\infty ))\right\}$$

and

$$z^{B}=lex\;\underset{x\in X}{\min}\left\{z_{2}(x),z_{1}(x):z(x)\in R((-\infty ,\infty ),(-\infty ,\infty ))\right\}$$

in parallel (Fig 6A). Then, the search space within *R*(***z***^*T*^,***z***^*B*^) is evenly constraint based on boundary conditions enforced w.l.o.g. on *z*_1_ (Fig 6B). The boundaries are calculated for a predefined number of constraints *m* with:

$$\tau_{i}^{z_{1}}=z_{1}^{T}+\frac{i∙(z_{1}^{B}-z_{1}^{T})}{m}\;with\;1\leq i\leq m.$$

Each section of the separated search space can be independently searched by solving $z^{i}=\mathrm{lex}\;\min_{x\in X}\;\left\{z_{1}(x),z_{2}(x):z(x)\in R({(\tau }_{i}^{z_{1}},z_{2}^{T}),z^{B})\right\}$ and the resulting new nondominated points can be used as initial approximation of the Pareto front. The found nondominated points might contain duplicates and might not resemble the complete Pareto front. Therefore, it is necessary to perform a refinement of the Pareto front to find the remaining nondominated points. To this end, the nondominated points are sorted in nondecreasing order such that $z_{1}^{1}\leq z_{1}^{2}\leq \cdots \leq z_{1}^{k}$. Each consecutive pair of points spans a search rectangle *R*(***z***^***i***^,***z***^***j***^) with *i* ≤ *j*. These rectangles can now be searched in parallel by the rectangle-splitting algorithm (Fig 6C). The search rectangles are split in half. First, the bottom half *R*^*B*^ is searched by solving:

$${\bar{z}}^{1}=lex\;\underset{x\in X}{\min}\;\{z_{1}\left(x\right),z_{2}\left(x\right):z\left(x\right)\in R((z_{1}^{i},\frac{z_{2}^{i}+z_{2}^{j}}{2}),z^{j})\}$$

If a nondominated point is found, the upper half *R*^*T*^ is further restricted and spans now $R\left(z^{i},({\bar{z}}_{1}^{i}‑\epsilon ,\frac{z_{2}^{i}+z_{2}^{j}}{2})\right)$ in which ${\bar{z}}^{2}=lex\;\min_{x\in X}\left\{z_{2}\left(x\right),z_{1}\left(x\right):z\left(x\right)\in R(z^{i},({\bar{z}}_{1}^{i}-\epsilon ,\frac{z_{2}^{i}+z_{2}^{j}}{2}))\right\}$ is searched. Each newly found point spans a new independent search rectangle $R(z^{i},{\bar{z}}^{2})$ and $R({\bar{z}}^{1},z^{j})$ with its adjacent point. These rectangles are searched in parallel with the described procedure (Fig 6D). If the search operation yielded the known point ***z***^*j*^ for $R\left((z_{1}^{i},\frac{z_{2}^{i}+z_{2}^{j}}{2}),z^{j}\right)$ and ***z***^*i*^ for $R\left(z^{i},({\bar{z}}_{1}^{i}‑\epsilon ,\frac{z_{2}^{i}+z_{2}^{j}}{2})\right)$ accordingly it proofs that the area does not contain further nondominated points. The search procedure is carried out until the complete search space has been explored.

### Inference of the maximum entropy sequence model

Multiple sequence alignments (MSA), created by JackHMMER[49], were used for the inference of the maximum entropy models of the Factor VIII C2-domain (residues 2,188–2,345 of FA8_HUMAN, Supplementary S1 Material). To optimize residue coverage and MSA diversity, the alignment was created using five search iterations at an E-value threshold of 10^−20^. Sequences with 70% or more gaps and columns with over 50% gaps were excluded from subsequent statistical inference. To reduce the influence of sampling bias in the inference step, sequences were clustered at a 90% identity threshold (theta 0.9), and reweighted by the inverse of the number of cluster members resulting in M_eff_ = 1656 effective sequences. Generally, at least a M_eff_/L ≥ 1 is considered necessary to predict the tertiary structure of a protein [31, 50]. Here we achieve a M_eff_/L ≥ 10 (with L = 157 aa), which should be sufficient to guarantee a high-quality model. The parameters of the pairwise maximum entropy model and evolutionary couplings were then inferred using EVfold^PLM^ [51] with pseudo-likelihood maximization [52]. Substitution effects were derived by calculating the difference between the wild-type and the mutant statistical energy [31]. The validity of the maximum entropy model was verified by using the precision of the inferred top evolutionary couplings (ECs) between residue pairs compared to an existing 3D structure. To identify the top ECs a Gaussian-lognormal mixture model was inferred based on the overall score distribution [28] and the ECs within the tail of the distribution (ECs with a probability ≥ 0.90 of belonging to the lognormal) were used for model quality assessment [28].

### Hemophilia a severity data and regression analysis

Single point mutation data with known patient severity status was extracted from the Factor VIII variant database (http://www.factorviii-db.org). The data was filtered for mutations residing within the C2 domain, which resulted in 40 data points in total (Supplementary S4 Table). The severity status of each patient was determined based on a one-stage Factor VIII:C and categorized into three classes–severe (<1%), moderate (1–5%), and mild (>5%). The data points were unevenly distributed across the classes with 15 severe, 8 moderate, and 17 mild cases.

To train and validate the multinomial and logistic regression models, the data was randomly divided into training and test set (70:30%-split) in a stratified manner. This process was repeated two hundred times and the prediction performance averaged over the runs.

### Experimental design

In order to experimentally verify our *in silico* predictions for Factor VIII, we utilized the commercial REVEAL HLA-Peptide binding assay of ProImmune (www.proimmune.com). Peptides were synthesized using the PEPscreen custom library synthesis method, yielding high purity peptides for experimental analysis. HLA-peptide binding was assessed for the three HLA-DRB1 alleles used in this study (DRB1*15:01, DRB1*03:01 and DRB1*07:01). In short, the method compares the affinity of the studied peptide to the affinity of a high-affinity control peptide. Each peptide is then scored for binding to a certain HLA molecule relative to the score of the control peptide and reported as the percentage of the signal generated by the control peptide.

### Implementation

The two-phase rectangle-splitting solver was implemented in Python 2.7 using the CPLEX package, Numpy 1.4, and Polygon 2.0.6 package. CPLEX 12.6 was used as backend to solve the BOMILP models.

Structure-based fitness prediction for validation purposes of the de-immunized Factor VIII C2 domain constructs were performed with FoldX [32] using default settings for the obtained mutations. TEPITOPEpan 1.0 was used for epitope prediction.

### Statistical analysis

The multinomial and logistic models were fit and evaluated using Scikit-learn 0.18 [53]. The statistical analysis was conducted with R 3.0.2. Statistical significance was considered at *α* = 0.05. The specific statistical tests used are indicated in the figures or in the results section.

## Supporting information

S1 Material The long-range evolutionary couplings of Factor VIII C2 domain, and the multiple sequence alignments used for model inference. (GZ)

S2 Material Detailed description of the de-immunization BOMILP formulation. (DOCX)

S1 Table Known Factor VIII C2 domain single point mutation with severity status and predicted statistical fitness change with the maximum entropy model and FoldX. (XLSX)

S2 Table Synthesized peptides sequences. (XLSX)

S3 Table Experimental immunogenicity score measured per peptide and HLA allele. (XLSX)

S4 Table Summarized experimental immunogenicity scores of the reconstructed immunogenic region. (XLSX)
