# Supplementary material for: Population-specific design of de-immunized protein biotherapeutics
Source: PLoS Comput Biol. 2018 Mar 2;14(3):e1005983. doi: 10.1371/journal.pcbi.1005983 (PMC5851651; doi:10.1371/journal.pcbi.1005983)
Supplement: S2 Material — (DOCX) [file pcbi.1005983.s002.docx]

Population-specific design of de-immunized protein biotherapeutics

Benjamin Schubert^1,2,3,4,*^, Charlotta Schärfe^1,2,4^, Pierre Dönnes^1,5^, Thomas Hopf^3,4^, Debora Marks^4,§^, and Oliver Kohlbacher^1,2,6,7,8,9,§^

^1^ Center for Bioinformatics, University of Tübingen, 72076 Tübingen, Germany

^2^ Applied Bioinformatics, Dept. of Computer Science, 72076 Tübingen, Germany

^3^ Department of Cell Biology, Harvard Medical School, Boston, 02115 Massachusetts, USA

^4^ Department of Systems Biology, Harvard Medical School, Boston, 02115 Massachusetts, USA

^5^ SciCross AB, 541 23 Skövde, Sweden

^7^ Quantitative Biology Center, 72076 Tübingen, Germany

^8^ Faculty of Medicine, University of Tübingen, 72076 Tübingen, Germany

^9^ Biomolecular Interactions, Max Planck Institute for Developmental Biology, 72076 Tübingen, Germany

^§^ Both authors contributed equally to this work.

^*^ Corresponding author:

E-mail: [benjamin_schubert@hms.harvard.edu](mailto:benjamin_schubert@hms.harvard.edu) (BS)

Table S6. Detailed description of the bi-objective mixed integer formulation used to solve the deimmunization problem.

| Definition: | |  |  | |  |
| --- | --- | --- | --- | --- | --- |
| Sets I: | |  |  | |  |
| $\boldsymbol{\Sigma}$ | |  | Amino acid alphabet | |  |
| $\boldsymbol{H}$ | |  | Set of HLA alleles | |  |
|  | |  |  | |  |
| Parameters I: | |  |  | |  |
| $\boldsymbol{n\in}\mathbb{N}_{\mathbf{>0}}$ | |  | The length of the sequence | |  |
| $\mathbf{e}_{\mathbf{n}}\boldsymbol{\in}\mathbb{N}_{\mathbf{>0}}$ | |  | The length of epitopes | |  |
| $\mathbf{k}\boldsymbol{\in} \mathbb{N}_{\mathbf{>0}}$ | |  | The induced mutation load | |  |
| $\mathbf{p}_{\boldsymbol{h}} \boldsymbol{\in}\mathbf{[0}\mathbf{,}\mathbf{1]}$ | | $h\in H$ | The HLA allele probability | |  |
| $\boldsymbol{\Phi}\mathbf{(}\mathbf{h,a,j}\mathbf{)}\boldsymbol{\in} \mathbb{R}$ | | $h\in H, a\in\Sigma, j \in\left\{ 0..e_{n}-1 \right\}$ | The TEPITOPEpan PSSM matrices | |  |
| $\boldsymbol{\tau}_{\boldsymbol{h}}\boldsymbol{\in}\mathbb{R}_{\boldsymbol{>0}}$ | | $h\in H$ | Binding threshold for allele $m\in A$ | |  |
|  | |  |  | |  |
| Set II: | |  |  | |  |
| $\mathbf{M}_{\mathbf{i}}\boldsymbol{\subseteq\Sigma}$ | | $i \in\{1..n\}$ | Set of possible variations per position | |  |
| $\boldsymbol{W}_{\boldsymbol{i}}\boldsymbol{\subseteq\Sigma}$ | | $i \in\{1..n\}$ | The WT sequence | |  |
|  | |  |  | |  |
| Parameters II: | |  |  | |  |
| $\mathbf{h}_{\boldsymbol{i,a}}\mathbb{\in R}$ | | $i\in\{1..n\}, a\in M_{i}$ | Inferred singleton fitness scores | |  |
| $\boldsymbol{J}_{\boldsymbol{i,j,a,b}}\mathbb{\in R}$ | | $1\leq i<j\leq n, a\in M_{i}, b\in M_{j}$ | Inferred coupling fitness scores | |  |
|  | |  |  | |  |
| Variables: | |  |  | |  |
| $\mathbf{x}_{\mathbf{i,a}}\mathbf{=1}$ | | $i\in\left\{ 1..n \right\}, a\in M_{i}$ |  | |  |
| $\mathbf{w}_{\mathbf{i,j,a,b}}\mathbf{=1}$ | | $1\leq i<j\leq n, a\in M_{i}, b\in M_{j}$ |  | |  |
|  | |  |  | |  |
| Objectives: | |  |  | |  |
| (O1) | $\mathrm{mi}n_{x,w} \sum_{h\in H} p_{h}\cdot\sum_{i=1}^{n-e_{n}} max\left( 0,\left( \sum_{j=0}^{e_{n}-1} \sum_{a\in M_{i+j}} x_{i+j,a}\cdot\phi\left( h,a,j \right) \right)-\tau_{h} \right)$ | | | Immunogenicity objective | |
| (O2) | $\mathrm{mi}n_{x,w} \sum_{i=1}^{n} {x_{i,a}\cdot h}_{i,a}+\sum_{i=1}^{n} \sum_{1\leq i<j\leq n} {w_{i,j,a,b}\cdot J}_{i,j,a,b}$ | | | Fitness objective | |
|  | |  |  | |  |
| Constraints: | |  |  | |  |
| (C1)$\sum_{\boldsymbol{a\in}\mathbf{M}_{\mathbf{i}}} \mathbf{x}_{\mathbf{i,a}}\mathbf{=1}$ | | $\forall i\in\{1..n\}$ | Ensures that only one AA is selected per position | |  |
| (C2) $\sum_{\boldsymbol{b\in}\mathbf{M}_{\mathbf{j}}} \mathbf{w}_{\mathbf{i,j,a,b=}\mathbf{x}_{\mathbf{i,a}}}$ | | $\forall i, a\in M_{i}, i>j\in\{1..n\}$ | Ensures that $w_{i,j,a,b}=1$ iff $x_{i,a}=1 \wedge x_{j,b}=1$ | |  |
| (C3) $\sum_{\boldsymbol{a\in}\mathbf{M}_{\mathbf{i}}} \mathbf{w}_{\mathbf{i,j,a,b=}\mathbf{x}_{\mathbf{j,b}}}$ | | $\forall j, b\in M_{i}, j<i\in\{1..n\}$ | Ensures that $w_{i,j,a,b}=1$ iff $x_{i,a}=1 \wedge x_{j,b}=1$ | |  |
| (C4) $\sum_{\mathbf{i=1}}^{\mathbf{n}} \sum_{\boldsymbol{a\in}\mathbf{W}_{\mathbf{i}}} \left( \mathbf{1-}\mathbf{x}_{\mathbf{i,a}} \right)\boldsymbol{\leq k}$ | |  | Ensures that only $k$ mutations are introduced | |  |
